# Gastrointestinal Basidiobolomycosis, a Rare and Under-diagnosed Fungal Infection in Immunocompetent Hosts: A Review Article

**Published:** 2015-03

**Authors:** Bita Geramizadeh, Mina Heidari, Golsa Shekarkhar

**Affiliations:** 1 Transplant Research Center, Nemazee Hospital, Shiraz University of Medical Sciences, Shiraz, Iran;; 2 Department of Pathology, School of Medicine, Shiraz University of Medical Sciences, Shiraz, Iran

**Keywords:** Gastrointestinal, Entomophthorales, Basidiobolus ranarum, Immunocompetent

## Abstract

Gastrointestinal Basidiobolomycosis (GIB) is an unusual, rare, but emerging fungal infection in the stomach, small intestine, colon, and liver.

It has been rarely reported in the English literature and most of the reported cases have been from US, Saudi Arabia, Kuwait, and Iran. In the last five years, 17 cases have been reported from one or two provinces in Iran, and it seems that it has been undiagnosed or probably unnoticed in other parts of the country.

In this review, we explored the English literature from 1964 through 2013 via PubMed, Google, and Google scholar using the following search keywords:

1. Basidiobolomycosis
2. Basidiobolus ranarum
3. Gastrointestinal Basidiobolomycosis

In this review, we attempted to collect all clinical, pathological, and radiological findings of the presenting patients; complemented with previous experiences regarding the treatment and prognosis of the GIB.

Since 1964, only 71 cases have been reported, which will be fully described in terms of clinical presentations, methods of diagnosis and treatment as well as prognosis and follow up.

## Introduction

Basidiobolomycosis is a rare fungal infection caused by Basidiobolus ranarum.^1^

The Zygomycetes includes two fungal orders: Mucorales and Entomophthorales, with completely different pathogenic potentials. Mucorales involve only the immunocompromised patient, while Entomophthorales, which include *Basidiobolus* genera, causes infection in immune competent individuals, mostly chronic infection of the subcutaneous tissue.^2^^-^^4^ This fungal infection has been extremely rare in immunocompromised hosts such as diabetics and renal transplant patients.^5^ Just the opposite, Mucormycosis has been rarely reported in immunocompetent hosts with no underlying disease.^6^^-^^8^

Basidiobolus ranarum (B. haptosporus, B. meristoporus) is a fungus belonging to the order Entomophthorales in the family of Zygomycota.^9^^,^^10^ Basidiobolus species are types of filamentous fungi belonging to the family of Basidiobolaceae of the order Entomophthorales.^11^^,^^12^ Basidiobolus ranarum was first described in 1886 in frogs. This fungus is an environmental saprophyte found in soil and decaying vegetable materials.^11^ This fungus is endemic in some parts of the world such as India.^13^^-^^15^

It is commonly found in soil and decaying vegetable materials and occasionally found as a commensal in the gastrointestinal tracts of amphibians, reptiles, fish, dogs, frogs, and bats.^16^^-^^18^ Even fatal cases have been reported in toads.^19^^,^^20^

B. ranarum is proven as a low virulent fungus. It has been identified in mice, i.e. after inoculation of this fungus in experimental mice; invasive disease has not occurred.^12^

The first recognized human case of infection caused by Basidiobolus ranarum was reported from Indonesia as a subcutaneous infection in 1956.^21^Since then, there have been few individual case reports regarding subcutaneous, nose, and sinus infection by this opportunistic fungus.^22^^,^^23^ Typically, this fungus causes chronic subcutaneous infection mainly in children as young as one month of age.^24^ Without treatment, the subcutaneous infection could be similar to soft tissue tumors; and thus, prompt treatment of the skin is essential.^25^ Even huge subcutaneous infections have been reported to cause perineal intestinal obstruction or intraperitoneal spread.^26^^,^^27^ Additionally, sometimes delayed treatment can cause disfigured extremities.^28^^,^^29^

It seems that, this fungal infection is acquired by exposure to B. ranarum after minor trauma to the skin or by biting insects.^30^ This rare fungus can also rarely infect other parts of the body especially gastrointestinal tract.^31^

In 1964, the first documented case of gastrointestinal Basidiobolomycosis (GIB) was reported in a 4-year-old boy.^17^ Thereafter, it was progressively identified and the number of case reports increased from different parts of the world such as Saudi Arabia, Kuwait, USA, and Iran. However, no definite and characteristic clinical presentation has been reported and all previous cases have been operated without initial diagnosis of GIB. Preoperative diagnosis has been different neoplastic and nonneoplastic diseases such as colorectal cancer and Crohn’s disease, inflammatory pseudotumor, etc.^11^

In this review, we extensively gathered information from previously reported cases in the literature with the emphasis on the demographic findings, presenting symptoms, laboratory findings, diagnostic tests, and treatment options. We conducted a comprehensive search of medical literature from 1964 through May 2014 via PubMed, Google, and Google scholar using the following search keywords:

1. Basidiobolomycosis
2. Basidiobolus ranarum
3. Gastrointestinal Basidiobolomycosis

All cases with the diagnosis of gastrointestinal Basidiobolomycosis in immunocompetent host were included in the review. Cases published more than once (duplicate case reports) were excluded. All important findings such as presenting symptoms, method of primary diagnosis, laboratory findings, operative findings, treatment modalities, and follow up studies were separately included in each case.

Since the first report of GIB in 1964 until the present, there have been 71 cases of GIB, in the English literature. Cases have been reported from Saudi Arabia with 23 cases,^1^^,^^11^^-^^17^^,^^31^^-^^37^ Iran with 17 cases,^38^^-^^41^ USA with 23 cases,^42^^-^^45^ Kuwait with 2 cases,^46^^-^^48^ The Netherlands with one case,^49^ and Iraq with 6 cases.^50^ The patients were ranged from 1.5 to 80 years old. This disease was significantly more common in males; from 71 reported cases, only six patients were female.

Nearly all patients had abdominal pain and fever as their initial symptoms; however, some cases additionally had constipation (4 cases), diarrhea (1 case), and gastrointestinal bleeding (1 case).^17^ Only one patient was presented with mucoid stool.^42^ Furthermore, one case was presented with perforated appendicitis because of fungal invasion in the appendiceal wall.^43^

Radiologic examination by ultrasonography and CT scan were reported in 20 cases as intestinal wall thickening in 8 and intestinal, gastric or abdominal masses in other 12 cases.^49^ There were 3 cases with preliminary diagnosis of inflammatory bowel disease such as Crohn’s disease with and without fistula.^12^^,^^35^^,^^36^^,^^45^ In 12 reported cases, there were concomitant liver and intestinal masses.^1^^,^^40^

Most cases had preoperative endoscopy and biopsy as diagnostic procedures, none of which led to any diagnoses. There were 3 cases with incorrect preliminary diagnosis of tuberculosis in the colon biopsy because of the presence of granuloma in the pathologic examination.^33^^,^^46^ The erythrocyte sedimentation rate (ESR) was 33 to 138, and eosinophil count was <1 to 27.7%. It is worthy to note that only in 5 cases eosinophil count was normal.^1^

The method of diagnosis was pathology and culture.^51^^,^^52^ In 32 cases, culture was performed, however only 50% turned out to be positive.

Pathologic diagnosis of the cases was characteristic and showed the same picture in all of them, i.e. the presence of Splendore-Hoeppli bodies and many eosinophils as well as intensely radiating eosinophilic granular material surrounding the fungal elements.^33^ This histological finding is very characteristic of this fungus and other invasive fungi with gastrointestinal involvement such as mucormycosis causes extensive necrosis and vascular invasion and even their granulomas are morphologically different with no eosinophil.^53^^,^^54^

Recently, there are reports of PCR (polymerase chain reaction) and molecular diagnosis of GIB, with high specificity and sensitivity.^55^ Also, there is a report of ultrastructural features of GIB. On ultrastructural level, fungal hyphae, spores, and macrophage-laden crystalloids could be seen.^56^

An extremely rare report on successful experimental diagnosis of this infection by immunodiffusion is published, but so far, no commercial test is available for immunologic diagnosis of B. ranarum infection.^57^

There are reports of elevated levels of cytokine responses such as IL4, IL10, TNFα (TH2 type cytokines) after infection with B. ranarum that can be helpful as a diagnostic test.^47^

Most of the cases (59 patients) undergone surgery with intestinal, and gastric or liver resection, and all cases received antifungal therapies including itroconazole and amphotericine B, but rarely ketoconazole, voriconazole, pesoconazole and fluconazole.^49^^,^^50^ There are a few reports on the successful treatment with Polymixin E.^58^

Among these 71 patients, eight cases deceased, probably due to delayed treatment and because of the disseminated disease. The deceased cases were pediatric except for one who was 61 years old.^34^ Five of the previously reported patients had recurrence, despite of fungal medical therapy and surgery.^1^^,^^46^^,^^50^ Table 1 shows the most common findings in the previously reported cases of GIB.

**Table 1:** Most common characteristics of gastrointestinal Basidiobolomycosis in previously reported cases

Reported findings	Most common characteristic
Presenting symptoms	Abdominal pain and fever
Age	1.5-80 years
Sex (M/F)	65/6
ESR	33-138
Eosinophils	1-27.7%
Most common preliminary diagnosis	Intestinal, gastric or liver mass
Culture positive cases	<50%
Pathologic diagnosis before surgery by endoscopy	0
Characteristic histological findings	100%
Recurrence	4 cases
Died	8 cases

## Discussion

There are more than 100,000 fungi worldwide, only 150 of which are pathogen.^32^ Basidiobolomycosis is a rare fungal infection due to *B. ranarum*, an environmental saprophyte. It is a member of the order Entomophthorales of the class Zygomycetes.^59^ B. ranarum is a well-described fungus in the skin and subcutaneous tissue; however, this fungal infection in the gastrointestinal tract is an emerging infection.^60^

It seems that most reported cases have been from parts of the world with warm climate, such as Arizona in the US, Fars province in Iran, Saudi Arabia, and Kuwait.^40^ We believe that this is due to the nature of the fungus, which mostly lives in a warm and humid environment and involves human GI tract by ingestion of contaminated fruits, vegetables, and water.^46^

Another important concern is the route of acquiring this infection. The proposed theories are ingestion of infected food (e.g. people with pica) and using contaminated papers for cleaning the skin (e.g. toilet papers). Some of the previous studies reported the infection years after a surgical procedure. Therefore, implanted fungus after surgery is another less likely theory.^47^ Another proposed theory is the history of ranitidine ingestion, which can decrease gastric acidity and allow fungal survival after gastric passing. In addition, Smoking can decrease mucosal WBC function and facilitate fungal infection by B. ranarum.^43^Previous reports from Arizona (USA) were on people working as gardener and landscaper. Consequently, work environment is another unproven exposure.^46^

Since the first case of GIB, about 71 cases have been reported most of which are during the last 10 years. It seems that preoperative diagnosis of GIB by radiologic and clinical features is relatively impossible. Most common imaging findings on this disease have been concentric intestinal, gastric wall thickening or polypoid mass where all are nonspecific findings.^61^The most important point that causes delayed diagnosis in GIB is its occurrence in immunocompetent host.^59^ This fungus involves immunocompetent and healthy hosts and all previously reported cases were on patients in good health except for a few reports on diabetic patients.^31^Conversely, most of fungal infections in GI tracts have been reported in the immunosuppressive patients. Thus, it is very difficult for the clinicians to think about a fungal infection in the GI tract of a completely healthy patient.^50^*B. ranarum* lies deep beneath the mucosa, so endoscopic biopsies are non-representative, and in none of the previously reported cases, the endoscopic biopsy was diagnostic.^34^

Morphology of B. *ranarum* shows hyphae and zygospores. Hyphae shows branching and septation with thin wall. The whole hyphae are surrounded with an eosinophilic and amorphous hyalinized material. Zygospores have foamy cytoplasm with a large nucleolus.^32^

Although histological features of this infection has been well characterized in the skin and subcutaneous tissue, but its presence in the GI tract is challenging for pathologists. Even, there were cases with the erroneous biopsy diagnosis of tuberculosis, simply due to the presence of granulomatous reaction the mucosa.^33^^,^^46^ In our unpublished experience, even in cases with preoperative biopsy, erroneous diagnoses of ameba, tuberculosis and even Crohn’s disease is very common.

The main histological findings of the pathologic sections stained with hematoxylin and eosin stain in gastrointestinal Basidiobolomycosis are as below:

1. Marked infiltration of eosinophils
2. Mixed infiltration of PMN leukocytes and granulomatous inflammation
3. Thin wall and broad hyphae surrounded by eosinophilic material, which are easily seen by hematoxylin and eosin staining, however Periodic Acid-Schiff (PAS) and Gomori Methenamine Silver (GMS) can intensify the fungal wall staining^43^
4. Zygospores which are very similar to trophozoites of amoebae^32^

This fungal infection has been misdiagnosed and underdiagnosed; the reason for such delayed diagnosis is multifactorial:

1. Nonspecific clinical manifestation
2. No definite risk factor
3. Negative endoscopy and colonoscopy (submucosal growth)^17^

The most common and best diagnostic clue for the diagnosis of this fungal infection is primarily by considering this disease in differential diagnosis. However, common presenting clinical and paraclinical findings in nearly all previously reported cases were the same; that is abdominal pain and fever in a patient with abdominal, gastrointestinal or colon mass or intestinal wall thickening who has concomitant significantly high ESR and eosinophilia.^62^

The gold standard for definite diagnosis of GIB is culture.^41^^,^^42^ For best results of the culture, the tissue should be inoculated to the culture media very soon, as it cannot survive at 4ºC.^60^ After 2 to 3 days of incubation in sabouraud agar at 20 to 30ºC, white to pale grey colonies with radial folds would appear.^31^

However, in most previous experiences, the patient has undergone surgery with the preoperative diagnosis of mass, cancer, or inflammatory bowel disease. Therefore, the specimen has been sent to a lab in formalin and no culture has been performed, however, even in cases with culture, negative results were common.^1^^,^^11^^,^^12^

The pathologic findings after surgery are characteristic, and in combination with clinical and laboratory tests can be diagnostic. Consequently, in the cases with no available culture or negative culture histopathologic findings can aid to avoid treatment delay.^40^

The only fungal infections, which are somehow similar in histology to *B. ranarum,* are *Conidiobolus coronatus, Conidiobolus* *incongruous,* and *Pythium insidiobolus*. These fungi are seen in the head and neck only in immunocompromised hosts.^31^ Viseral involvement of these fungi has not been reported.^45^

Immunologic diagnosis of B. *ranarum* with methods such as immunodiffusion has been reported, which seems to be specific and has no cross reactivity with other fungi of Entomophthorales; however, its sensitivity is controversial. This immunodiagnostic test can also be helpful for follow up of patients.^43^

Molecular diagnosis has also been performed in some reports with optimum results by DNA extracted from formalin fixed paraffin embedded tissue, however, due to the rarity of the disease; many centers do not have the set up for this method.^63^

Another important concern with this fungal infection is prompt treatment by combined surgery and medical therapy to eradicate disease and prevent early recurrence. Delayed treatment can cause disseminated disease, which has life-threatening outcome and causes postmortem diagnosis after autopsy studies.^64^ Additionally, delayed treatment can cause complications such as bowel perforation, obstructive uropathy, esophageal varices, and duodenobiliary fistula.^1^

It seems that overall diagnosis of fungal infections is increasing even in immunocompetent hosts. Fortunately, there has been an increase in the number of broad-spectrum antifungal agents allowing for better therapeutic choices.^65^^-^^67^

On this specific fungus (i.e. Basidiobolus ranarum), there have been diverse experiences about the choice of antifungal therapy. Even rare reports on medicinal plants indicate its success as a form of treatment.^68^ Very few studies have reported successful treatment with amphotericin B, though with resistance in more than 50% of the treated cases.^33^ It seems that the best regimens involve pre- and postoperative itroconazole for at least 6 months. Prolonged treatment can prevent recurrence, but early discontinuation of the antifungal therapy is reported to cause recurrence of the disease even after surgery.^44^ Until now, no fungal resistance has been reported by itroconazole.^69^ Recently, another new antifungal with successful treatment with posaconazole is reported.^64^^,^^70^

## Conclusion

In this review, gastrointestinal Basidiobolomycosis is introduced as an emerging infection, and its life threatening and aggressive nature is emphasized. It requires prompt diagnosis and surgery; otherwise, it can be fatal with delayed and inadequate treatment.

In addition, there are unresolved questions in this fungal infection, the most important of which is predisposing factors for its acquisition in healthy people. This question needs more experience and diagnosing more cases to find out the exact predisposing factors. 

## References

[1] Al-Shanafey S, AlRobean F, Bin Hussain I (2012). Surgical management of gastrointestinal Basidiobolomycosis in pediatric patients. J Pediatr Surg.

[2] Mohta A, Neogi S, Das S (2011). Gastrointestinal mucormycosis in an infant. Indian J Pathol Microbiol.

[3] Chakrabarti A, Chatterjee SS, Das A, Panda N, Shivaprakash MR, Kaur A (2009). Invasive zygomycosis in India: experience in a tertiary care hospital. Postgrad Med J.

[4] Mantadakis E, Samonis G (2009). Clinical presentation of zygomycosis. Clin Microbiol Infect.

[5] Choonhakarn C, Inthraburan K (2004). Concurrent subcutaneous and visceral basidiobolomycosis in a renal transplant patient. Clin Exp Dermatol.

[6] Choi HL, Shin YM, Lee KM, Choe KH, Jeon HJ, Sung RH (2012). Bowel infarction due to intestinal mucormycosis in an immunocompetent patient. J Korean Surg Soc.

[7] Shiva Prasad BN, Shenoy A, Nataraj KS (2008). Primary gastrointestinal mucormycosis in an immunocompetent person. J Postgrad Med.

[8] Carr EJ, Scott P, Gradon JD (1999). Fatal gastrointestinal mucormycosis that invaded the postoperative abdominal wall wound in an immunocompetent host. Clin Infect Dis.

[9] Anaparthy UR, Deepika G (2014). A case of subcutaneous zygomycosis. Indian Dermatol Online J.

[10] Kumar Verma R, Shivaprakash MR, Shanker A, Panda NK (2012). Subcutaneous zygomycosis of the cervicotemporal region: Due to Basidiobolus ranaram. Med Mycol Case Rep.

[11] Al-Shanafey S, AlRobean F, Bin Hussain I (2012). Surgical management of gastrointestinal basidiobolomycosis in pediatric patients. J Pediatr Surg.

[12] El-Shabrawi MH, Kamal NM, Jouini R, Al-Harbi A, Voigt K, Al-Malki T (2011). Gastrointestinal Basidiobolomycosis: an emerging fungal infection causing bowel perforation in a child. J Med Microbiol.

[13] Jayanth ST, Gaikwad P, Promila M, Muthusami JC (2013). The Sinus That Breeds Fungus: Subcutaneous Zygomycosis Caused by Basidiobolus ranarum at the Injection Site. Case Rep Infect Dis.

[14] Sujatha S, Sheeladevi C, Khyriem AB, Parija SC, Thappa DM (2003). Subcutaneous zygomycosis caused by Basidiobolus ranarum - a case report. Indian J Med Microbiol.

[15] Maiti PK, Bose R, Bandyopadhyay S, Bhattacharya S, Dey JB, Ray A (2004). Entomophthoromycosis in South Bengal (Eastern India): a 9 years study. Indian J Pathol Microbiol.

[16] Saadah OI, Farouq MF, Daajani NA, Kamal JS, Ghanem AT (2012). Gastrointestinal basidiobolomycosis in a child; an unusual fungal infection mimicking fistulising Crohn’s disease. J Crohns Colitis.

[17] Rabie ME, El Hakeem I, Al-Sharim M, Al Skini MS, Jamil S (2011). Basidiobolomycosis of the colon masquerading as stenotic colon cancer. Case Rep Surg.

[18] Paré JA (2003). Fungal diseases of amphibians: an overview. Vet Clin North Am Exot Anim Pract.

[19] Taylor SK, Williams ES, Mills KW (1999). Mortality of captive Canadian toads from Basidiobolus ranarum mycotic dermatitis. J Wildl Dis.

[20] Taylor SK, Williams ES, Mills KW (1999). Experimental exposure of Canadian toads to Basidiobolus ranarum. J Wildl Dis.

[21] Kian Joe L, Pohan A, Tjoei ENG, Van Der Meulen H (1956). Basidiobolus ranarum as a cause of subcutaneous mycosis in Indonesia. AMA Arch Derm.

[22] Goyal A, Gupta N, Das S, Jain S (2010). Basidiobolomycosis of the nose and face: a case report and a mini-review of unusual cases of basidiobolomycosis. Mycopathologia.

[23] Mathew R, Kumaravel S, Kuruvilla S, Varghese RG, Shashikala S, Srinivasan S (2005). Successful treatment of extensive basidiobolomycosis with oral itraconazole in a child. Int J Dermatol.

[24] Mendiratta V, Karmakar S, Jain A, Jabeen M (2012). Severe cutaneous zygomycosis due to Basidiobolus ranarum in a young infant. Pediatr Dermatol.

[25] Anand M, Deshmukh SD, Pande DP, Naik S, Ghadage DP (2010). Subcutaneous Zygomycosis Due to Basidiobolus ranarum: A Case Report from Maharastra, India. J Trop Med.

[26] Radjou AN, Rajesh NG (2011). Intestinal obstruction due to Basidiobolus ranarum: an unusual case. Indian J Med Microbiol.

[27] Vianna LM, de Lacerda MV, de Moraes MA (2005). Case report of subcutaneous entomophthoromycosis with retroperitoneal invasion. Rev Soc Bras Med Trop.

[28] Ramesh V, Ramam M, Capoor MR, Sugandhan S, Dhawan J, Khanna G (2010). Subcutaneous zygomycosis: report of 10 cases from two institutions in North India. J Eur Acad Dermatol Venereol.

[29] Singh R, Xess I, Ramavat AS, Arora R (2008). Basidiobolomycosis: a rare case report. Indian J Med Microbiol.

[30] Gugnani HC (1999). A review of zygomycosis due to Basidiobolus ranarum. Eur J Epidemiol.

[31] Nemenqani D, Yaqoob N, Khoja H, Al Saif O, Amra NK, Amr SS (2009). Gastrointestinal basidiobolomycosis: an unusual fungal infection mimicking colon cancer. Arch pathol Lab Med.

[32] Hussein MR, Musalam AO, Assiry MH, Eid RA, El Motawa AM, Gamel AM (2007). Histologic and ultrastructural features of gastrointestinal Basidiobolomycosis. Mycol Res.

[33] Wasim Yusuf N, Assaf HM, Rotowa NA (2003). Invasive gastrointestinal Basidiobolus ranarum infection in an immunocompetent child. Pediatr Infect Dis J.

[34] Al Jarie A, Al-Mohsen I, Al Jumaah S, Al Hazmi M, Al Zamil F, Al Zahrani M (2003). Pediatric gastrointestinal basibolomycosis. Pediatr Infect Dis J.

[35] Alshehri AH, Alshehri A, Bawahab MA, Al-Humayed S, Nabrawi K, Alamri FS (2013). Basidiobolomycosis: an emerging fungal infection of the gastrointestinal tract in adults. AJID.

[36] Al-Asmi MM, Faqeehi HY, Alshahrani DA, Al-Hussaini AA (2013). A case od pediatric gastrointestinal Basidiobolomycosis mimicking Crohn’s disease. A review of pediatric literature. Saudi Med J.

[37] Yusuf NW, Assaf HM, Rotowa NA (2003). Invasive Gastrointestinal Basidiobolus Ranarum Infection in an Immunocompetent Child. Pediatr Infect Dis J.

[38] AlSaleem K, Al-Mehaidib A, Banemai M, bin-Hussain I, Faqih M, Al Mehmadi (2013). Gastrointestinal Basidiobolomycosis: mimicking Crohn’s disease case report and review of the literature. Ann Saudi Med.

[39] Zahir ST, Shararhjin NS, Kargar S (2013). Basidiobolomycosis a mysterious fungal infection mimic small intestinal and colonic tumour with renal insufficiency and ominous outcome. BMJ case Rep.

[40] Arjmand R, Karimi A, Sanaei Dashti A, Kadivar M (2012). A child with intestinal Basidiobolomycosis. Iran J Med Sci.

[41] Zabolinejad N, Naseri A, Davoudi Y, Joudi M, Aelami MH (2014). Colonic basidiobolomycosis in a child: report of a culture-proven case. Int J Infect Dis.

[42] Geramizadeh B, Foroughi R, Keshtkar-Jahromi M, Malek-Hosseini SA, Alborzi A (2012). Gastrointestinal Basidiobolomycosis, an emerging infection in the immunocompetent host: a report of 14 patients. J Med Microbiol.

[43] Lyon GM, Smilack JD, Komatsu KK, Pasha TM, Leighton JA, Guarner J (2001). Gastrointestinal Basidiobolomycosis in Arizona: clinical and epidemiological characteristics and review of the literature. Clin Infect Dis.

[44] Pandit V, Rhee P, Aziz H, Jehangir Q, Friese RS, Joseph PH (2014). Perforated appendicitis with gastrointestinal Basidiobolomycosis: a rare finding. Surg Infect (Larchmt).

[45] Zavasky DM, Samowitz W, Loftus T, Segal H, Carroll K (1999). Gastrointestinal zygomycotic infection caused by Basidiobolus ranarum: case report and review. Clin Infect Dis.

[46] Vikram HR, Smilack JD, Leighton JA, Crowell MD, De Petris G (2012). Emergence of gastrointestinal Basidiobolomycosis in the united states, with a review of worldwide cases. Clin Infect Dis.

[47] Khan ZU, Khoursheed M, Makar R, Al-Waheeb S, Al-Bader I, Al-Muzaini A (2001). Basidiobolus ranarum as an etiologic agent of gastrointestinal zygomycosis. J Clin Microbiol.

[48] Khan ZU, Chugh TD (2000). Invasive fungal infections in Kuwait: A retrospective study. Indian J Chest Dis Allied Sci.

[49] Khan Zu, Prakash B, Kapoor MM, Madda JP, Chandy R (1998). Basidiobolomycosis of the rectum masquerading as Crohn’s disease: case report and review. Clin Infect Dis.

[50] van der Berk GE, Noorduyn LA, van Ketel RJ, van Leeuwen J, Bemelman WA, Prins JM (2006). A fatal pseudo-tumor: disseminated Basidiobolomycosis. BMC Infect Dis.

[51] Mishra P, Kshirsagar PR, Nilegaonkar SS, Singh SK (2012). Statistical optimization of medium components for production of extracellular chitinase by Basidiobolus ranarum: a novel biocontrol agent against plant pathogenic fungi. J Basic Microbiol.

[52] Shetty S, Mambatta AK, Penmatsa KR, Venkatakrishnan L (2014). Ileal mucormycosis: a rare cause of lower gastrointestinal bleeding. Ann Gastroenterol.

[53] Goel P, Jain V, Sengar M, Mohta A, Das P, Bansal P (2013). Gastrointestinal mucormycosis: a success story and appraisal of concepts. J Infect Public Health.

[54] Lalwani S, Govindasamy M, Gupta M, Siraj F, Varma V, Mehta N (2012). Gastrointestinal mucormycosis--four cases with different risk factors, involving different anatomical sites. Indian J Gastroenterol.

[55] Gómez-Muñoz MT, Fernández-Barredo S, Martínez-Díaz RA, Pérez-Gracia MT, Ponce-Gordo F (2012). Development of a specific polymerase chain reaction assay for the detection of Basidiobolus. Mycologia.

[56] Hussein MR, Musalam AO, Assiry MH, Eid RA, El Motawa AM, Gamel AM (2007). Histological and ultrastructural features of gastrointestinal Basidiobolomycosis. Mycol Res.

[57] Imwidthaya P, Srimuang S (1992). Immunodiffusion test for diagnosing basidiobolomycosis. Mycopathologia.

[58] Goel P, Jain V, Sengar M, Mohta A, Das P, Bansal P (2013). Gastrointestinal mucormycosis: a success story and appraisal of concepts. J Infect Public Health.

[59] Hassan HA, Majid RA, Rashid NG, Nuradeen BE, Abdulkarim QH, Hawramy TA (2013). Eosinophilic granulomatous gastrointestinal and hepatic abscesses attributable to Basidiobolomycosis and fascioliasis: a simultaneous emergence in Iraqi Kurdistan. BMC Infect Dis.

[60] Bigliazzi C, Poletti V, Dell’Amore D, Saragoni L, Colby TV (2004). Disseminated Basidiobolomycosis in an immunocompetent woman. J Clin Microbiol.

[61] Nguyen BD (2000). CT features of basidiobolomycosis with gastrointestinal and urinary involvement. AJR Am J Roentgenol.

[62] Geramizadeh B, Modjalal M, Nabai S, Banani A, Forootan HR, Hooshdaran F (2007). Gastrointestinal zygomycosis: a report of three cases. Mycopathologia.

[63] Rose SR, Lindsley MD, Hurst SF, Paddock CD, Damodaran T, Bennett J (2012). Gastrointestinal basidiobolomycosis treated with posaconazole. Med Mycol Case Rep.

[64] Al-Shanafey S, AlRobean F, Bin Hussain I (2012). Surgical management of gastrointestinal basidiobolomycosis in pediatric patients. J Pediatr Surg.

[65] Al-Abdely HM (2004). Management of rare fungal infections. Curr Opin Infect Dis.

[66] Clark TA, Hajjeh RA (2002). Recent trends in the epidemiology of invasive mycoses. Curr Opin Infect Dis.

[67] Warnock DW (2007). Trends in the epidemiology of invasive fungal infections. Nihon Ishinkin Gakkai Zasshi.

[68] Nwosu MO, Okafor JI (1995). Preliminary studies of the antifungal activities of some medicinal plants against Basidiobolus and some other pathogenic fungi. Mycoses.

[69] Richardson MD (2005). Changing patterns and trends in systemic fungal infections. J Antimicrob Chemother.

[70] El-Shabrawi MH, Kamal NM (2011). Gastrointestinal Basidiobolomycosis in children: an overlooked emerging infection?. J Med Microbiol.

